# Supplementary material for: AT2-intrinsic Z-AAT expression drives conserved inflammatory and proteotoxic stress responses and predisposes to emphysema
Source: bioRxiv. 2026 Apr 8:2026.04.06.715869. Preprint. [Version 1] doi: 10.64898/2026.04.06.715869 (PMC13082089; doi:10.64898/2026.04.06.715869)
Supplement: Supplement 1 [file media-1.pdf]

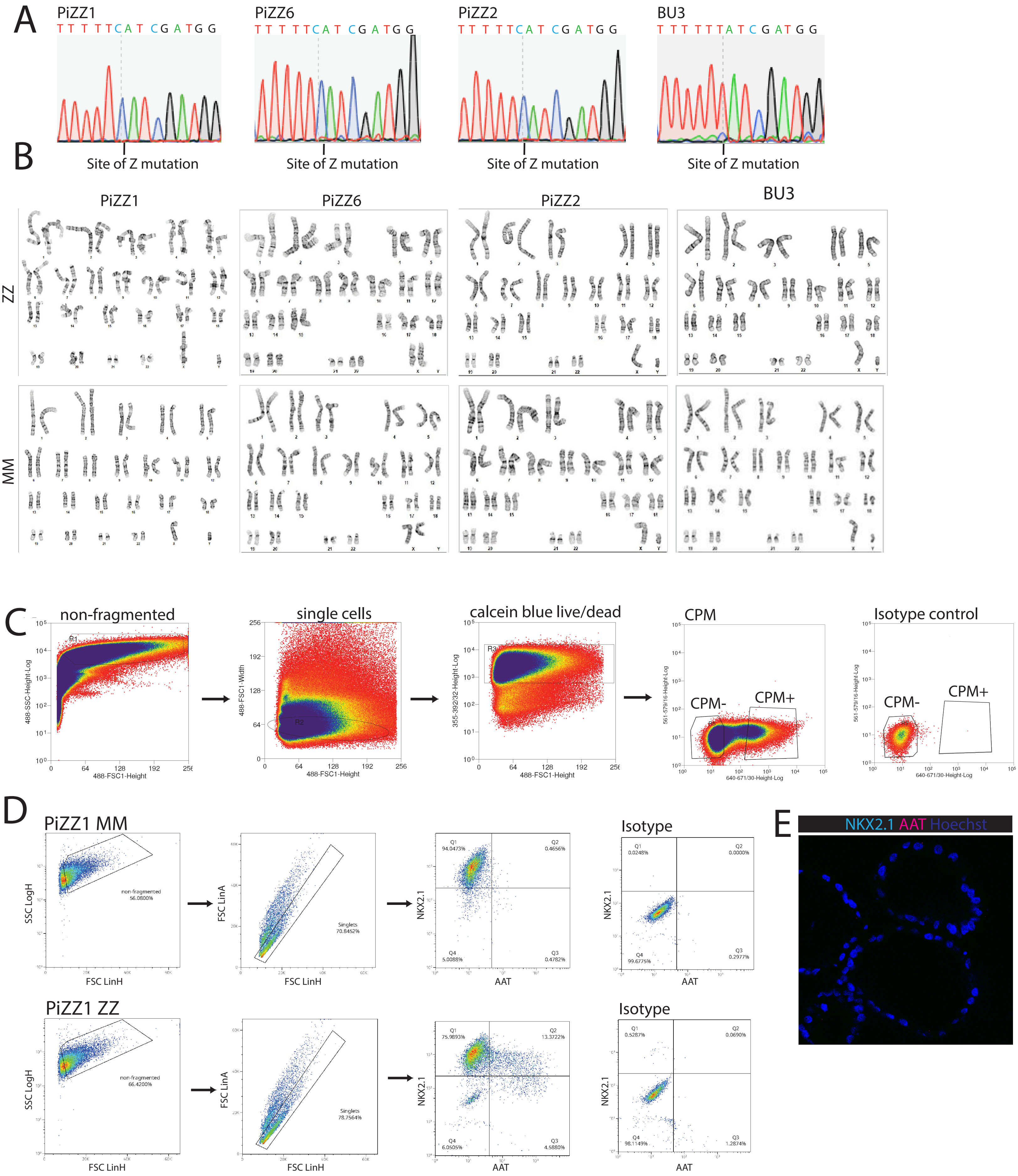
A
PiZZ1
PiZZ6
PiZZ2
BU3
Site of Z mutation
B
ZZ
MM
C
non-fragmented
single cells
calcein blue live/dead
CPM
Isotype control
CPM-
CPM+
488-SSC-Height-Log
488-FSC1-Height
488-FSC1-Width
355-392/32-Height-Log
561-579/16-Height-Log
640-671/30-Height-Log
D
PiZZ1 MM
PiZZ1 ZZ
Isotype
SSC LogH
FSC LinH
FSC LinA
NKX2.1
AAT
E
NKX2.1 AAT Hoechst

Figure S1

A) Sanger sequencing of iPSC lines after CRISPR-editing.
B) Karyotypes of iPSC lines before and after CRISPR-editing.
C) Representative flow plots and gating of d14 CPM sort in iAT2 differentiation.
D) Representative flow plots and gating for NKX2.1 and AAT staining and isotype controls.
E) Isotype control immunofluorescence staining for NKX2.1 and AAT protein.

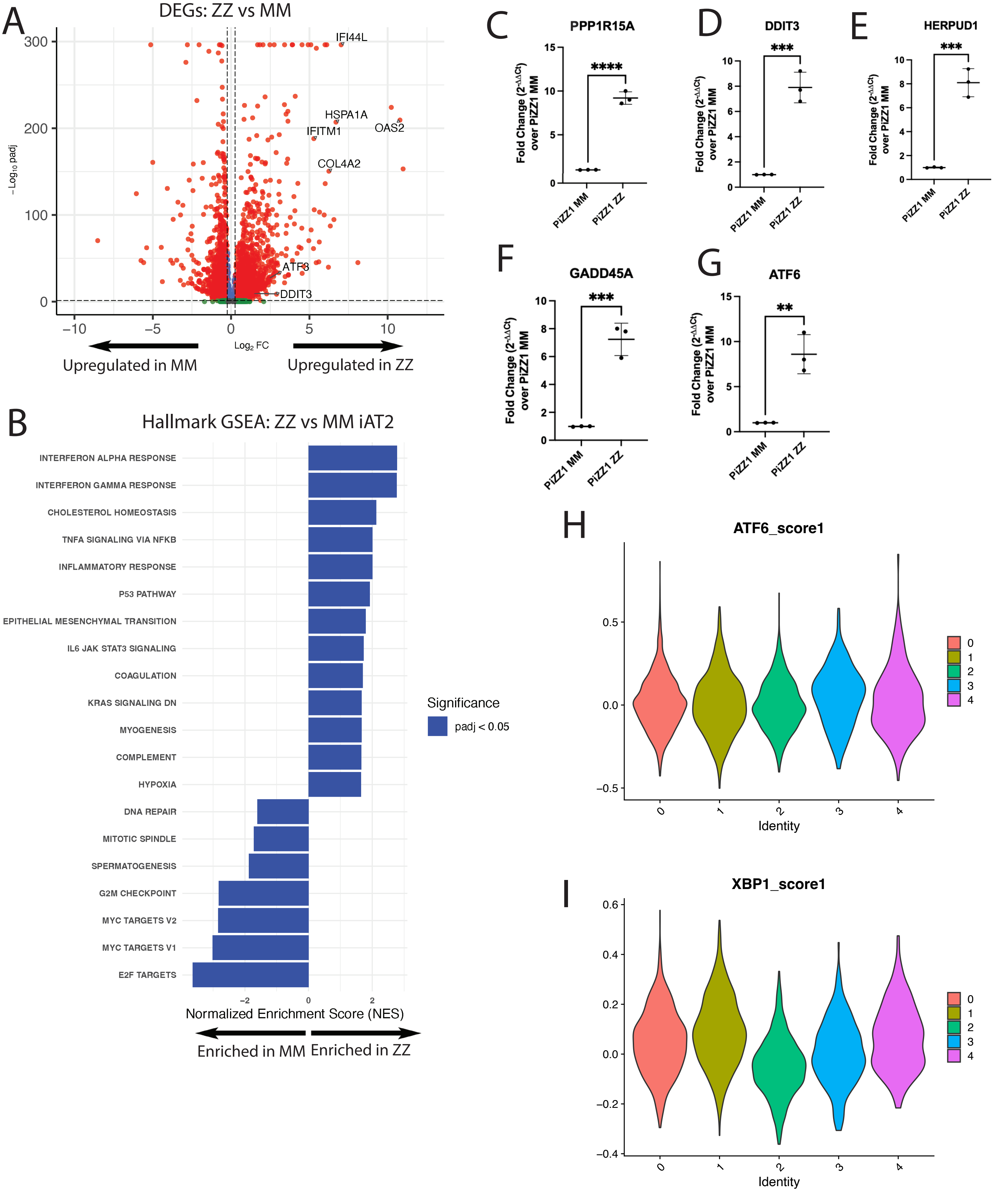
A
DEGs: ZZ vs MM
IFI44L
HSPA1A
IFITM1
OAS2
COL4A2
ATF3
DDIT3
Log2 FC
Upregulated in MM
Upregulated in ZZ
B
Hallmark GSEA: ZZ vs MM iAT2
INTERFERON ALPHA RESPONSE
INTERFERON GAMMA RESPONSE
CHOLESTEROL HOMEOSTASIS
TNFA SIGNALING VIA NFKB
INFLAMMATORY RESPONSE
P53 PATHWAY
EPITHELIAL MESENCHYMAL TRANSITION
IL6 JAK STAT3 SIGNALING
COAGULATION
KRAS SIGNALING DN
MYOGENESIS
COMPLEMENT
HYPOXIA
DNA REPAIR
MITOTIC SPINDLE
SPERMATOGENESIS
G2M CHECKPOINT
MYC TARGETS V2
MYC TARGETS V1
E2F TARGETS
Significance
padj < 0.05
Normalized Enrichment Score (NES)
Enriched in MM
Enriched in ZZ
C
PPP1R15A
D
DDIT3
E
HERPUD1
F
GADD45A
G
ATF6
Fold Change (2-ΔΔCt) over PiZZ1 MM
PiZZ1 MM
PiZZ1 ZZ
H
ATF6_score1
I
XBP1_score1
Identity

Figure S2
A)Volcano plot of DEGs comparing ZZ versus MM iAT2s by scRNA-seq. Dashed lines show padj < .05 and log2FC > .25. A subset of upregulated genes is highlighted with large font.
B)Hallmark GSEA of ZZ versus MM iAT2s ranked by normalized enrichment score (NES).
C-G) qRT-PCR of select genes upregulated in scRNA-seq from a separate differentiation. PiZZ1 line, n=3 technical replicates. Data represented as mean +/- SD. **p < 0.01, ***p < 0.001, ****p < 0.0001 by Student's T test.
H) Violin plot of ATF6 module score across all iAT2 clusters.
I) Violin plot of XBP1 module score across all iAT2 clusters.

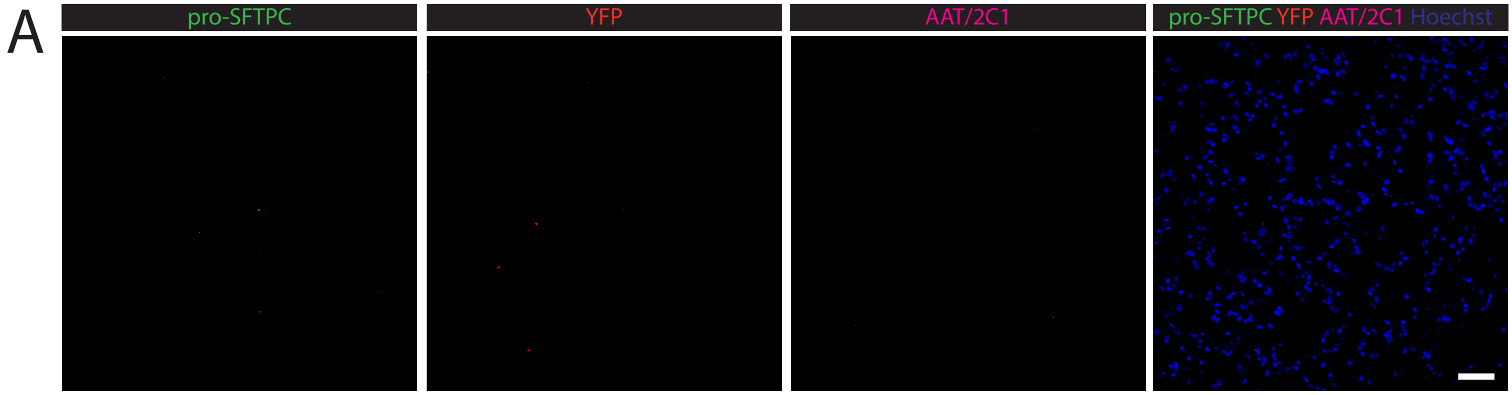

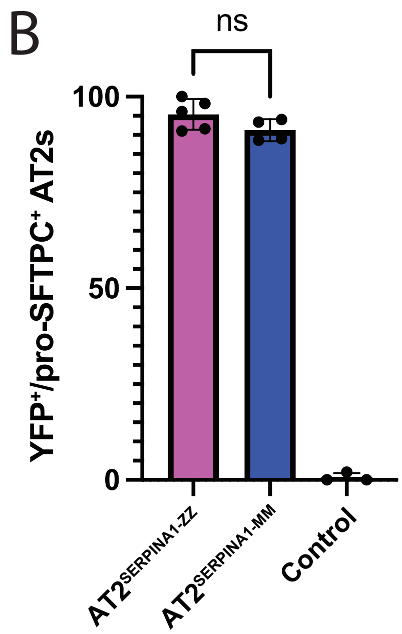

Figure S3
A) Isotype control immunofluorescence staining for pro-SFTPC, eYFP, and AAT/2C1 protein.
B) Mean percent of pro-SFTPC+ iAT2s staining positive for eYFP in AT2SERPINA1-ZZ, AT2SERPINA1-MM, and control mice. Each dot represents the mean across multiple fields of view from one mouse. Data represented as mean +/- SD.

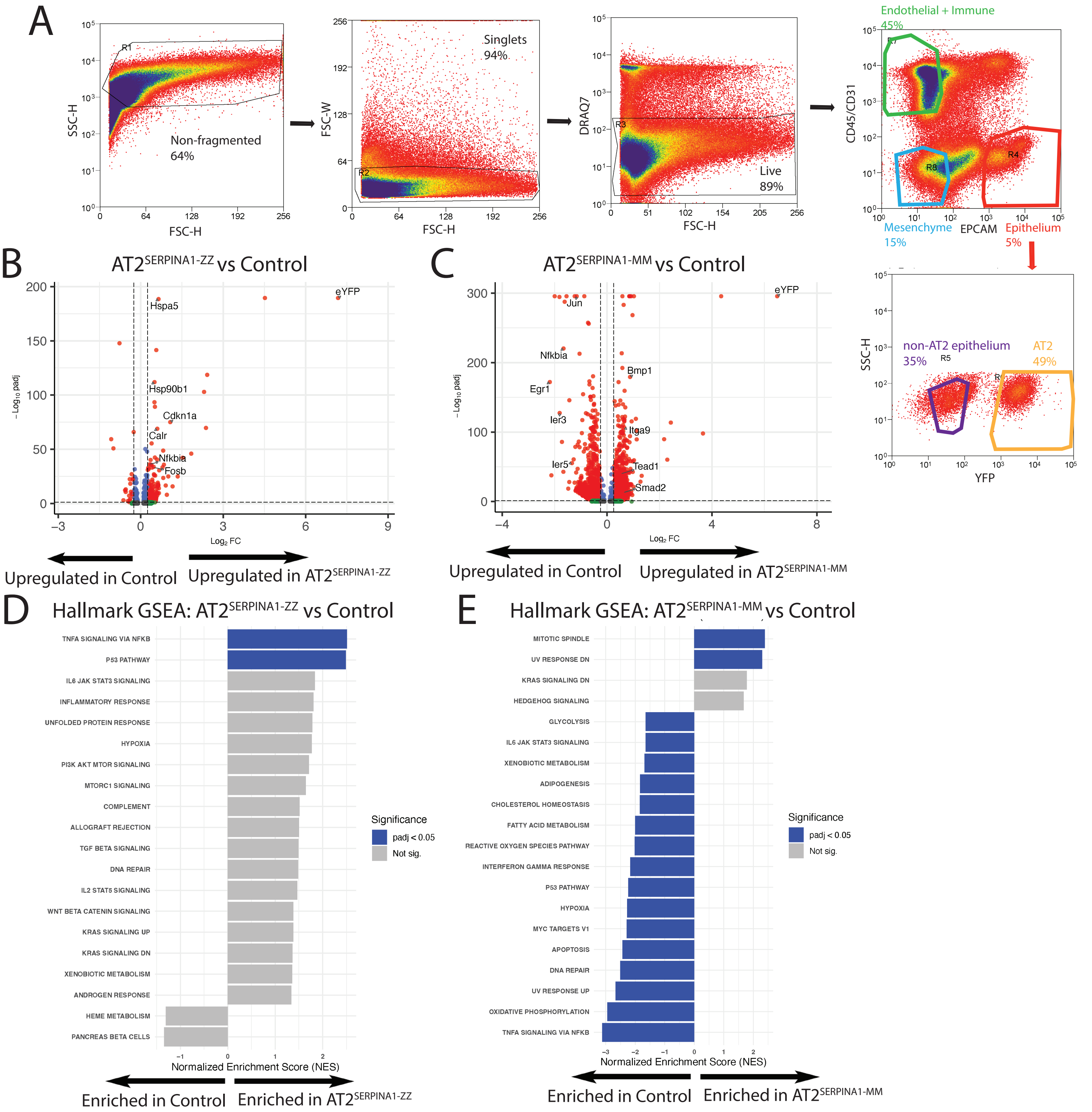

Figure S4

A) Representative sort plots for isolating live endothelial, immune, mesenchymal, epithelial, and eYFP+ from mouse lung for scRNA-seq.

B/C) Volcano plot of DEGs comparing B) AT2SERPINA1-ZZ AT2s versus control AT2s and C) AT2SERPINA1-MM AT2s versus control AT2s by scRNA-seq. Dashed lines show padj < .05 and log2FC > .25. A subset of enriched genes is highlighted with large font.

D/E) Hallmark GSEA of D) AT2SERPINA1-ZZ AT2s versus control AT2s and E) AT2SERPINA1-MM AT2s versus control AT2s ranked by normalized enrichment score (NES).

A

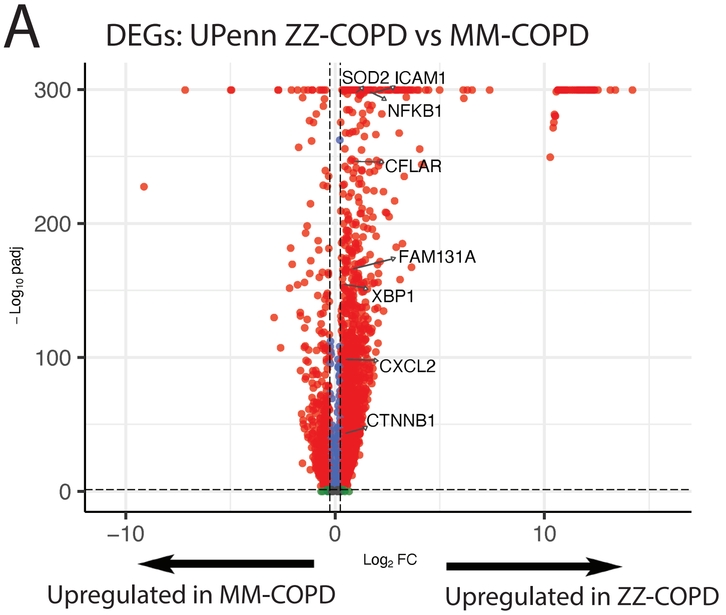

Figure S5

A) Volcano plot of differentially expressed genes (DEGs) comparing UPenn ZZ-COPD AT2s versus wild-type MM-COPD AT2s by scRNA-seq. Dashed lines show padj < .05 and log2FC > .25. A subset of upregulated genes is highlighted with large font.

A GO:BP EnrichR: Shared Genes iAT2s and Mouse (324 genes)

Term	Padj	OR	Select Genes
**Negative regulation of programmed cell death**	2.0E-5	4.32	XBP1, ANXA1, DNAJA1, KRT18, HMOX1, IER3
**Response to endoplasmic reticulum stress**	2.0E-05	6.03	PPP1R15A, JUN, XBP1, HERPUD1, DDIT3, ATF4
**Response to unfolded protein**	2.0E-05	16.03	DNAJA1, DDIT3, HERPUD1, PTPN1, DNAJB4, DNAJB2
**Regulation of apoptotic process**	2.5E-05	3.21	LGALS3, GADD45A, ATF4, XBP1, JUN, ANXA1
**Negative regulation of apoptotic process**	1.1E-04	3.57	IER3, XBP1, ANXA1, SOD2, DNAJA1, NUPR1
**Integrated stress response signaling**	3.5E-04	16.07	CEBPB, JUN, CEBPD, DDIT3, FOS, ATF4
Positive regulation of catabolic process	5.3E-04	5.21	PTPN1, HERPUD1, CDKN1B, MSN, XBP1, TRIM8
**Positive regulation of autophagy**	1.2E-03	7.01	TRIM8, CDKN1B, HERPUD1, NFE2L2, XBP1, BNIP3
Negative regulation of DNA-templated transcription	1.7E-03	2.41	CEBPB, CDKN1B, XBP1, JUN, GADD45A, ATF4
Positive regulation of intracellular signal transduction	1.7E-03	2.77	PPP1R15A, TRIM8, HMOX1, XBP1, DDIT3, REL

B GO:BP EnrichR: Shared Genes Primary AT2s and Mouse (592 genes)

Term	Padj	OR	Select Genes
Positive regulation of DNA-templated transcription	3.5E-13	2.92	IER2, MAP2K3, JUN, XBP1, JUND, NFKBIA
Positive regulation of transcription by RNA polymerase II	6.1E-12	3.05	AREG, IER2, RELA, JUN, REL, FOS
**Regulation of apoptotic process**	5.1E-10	3.22	IER3, ANXA1, TNFRSF12A, RELA, HSPA5, CALR
**Negative regulation of apoptotic process**	4.8E-7	3.25	ICAM1, SOD2, NFKB1, DNAJA1, KRT18, BIRC3
**Negative regulation of programmed cell death**	4.8E-7	3.57	HMOX1, IER3, XBP1, TP53, RELA, BIRC3
Negative regulation of DNA-templated transcription	4.8E-7	2.47	NFKB1, SMAD7, CALR, JUN, JUND, ATF3
Regulation of transcription by RNA polymerase II	4.9E-7	1.97	HMOX1, GADD45A, REL, CALR, EIF4A1, JUN
Negative regulation of transcription by RNA polymerase II	6.5E-7	2.71	AREG, TP53, ATF3, JUN, XBP1, GADD45A
**Response to endoplasmic reticulum stress**	1.3E-6	4.73	PTPN1, JUN, XBP1, HSPA5, CALR, ATF6
**Regulation of protein ubiquitination**	1.6E-6	6.38	HSP90AB1, TNFAIP3, DNAJB2, DNAJA1, BIRC2, BIRC3

C GO:BP EnrichR: Shared Genes Primary AT2s and iAT2s (905 genes)

Term	Padj	OR	Select Genes
Positive regulation of DNA-templated transcription	7.9E-13	2.48	ATF3, JUN, XBP1, KLF6, JUND, JUP
Positive regulation of transcription by RNA polymerase II	7.2E-11	2.53	HIF1A, JUND, JUP, VEGFA, LIF, XBP1
Regulation of transcription by RNA polymerase II	2.5E-8	1.86	HSPA1A, KLF6, VEGFA, HMOX1, JUN
Regulation of DNA-templated transcription	5.9E-6	1.74	HIF1A, JUND, NCOA2, HMOX1, KLF6, BCL3
Negative regulation of DNA-templated transcription	5.9E-6	2.07	CTNND1, GADD45A, HSPA1A, HIF1A, XBP1, JUN
Regulation of gene expression	1.3E-5	1.97	NFKBIZ, REL, HSPA1A, HSPA1B, CCNL2, HIF1A
Negative regulation of cell motility	3.2E-5	4.27	SERPINB1, PTEN, DUSP10, CYP1B1, ARID4A, FOXO3
Regulation of cell migration	3.7E-5	2.48	ARID4B, DUSP10, CASP8, CYP1B1, SERPINB1, ANXA1
Negative regulation of transcription by RNA polymerase II	6.0E-5	2.14	CTNND1, ATF3, JUN, XBP1, KLF4, FOSB
Negative regulation of cell migration	3.9E-4	3.38	PTEN, ARID4A, ARID4B, DUSP10, SERPINB1, HMOX1

D NF-kB Module Score Mouse

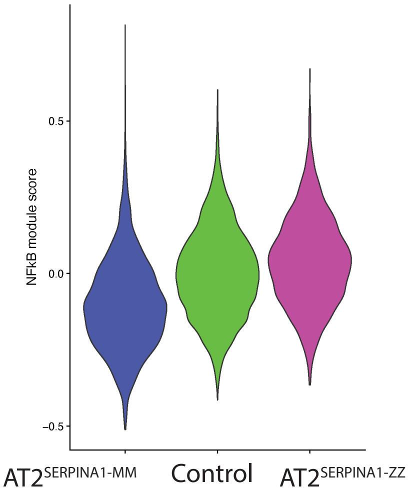

E Inflammatory AT2 Module Score Mouse

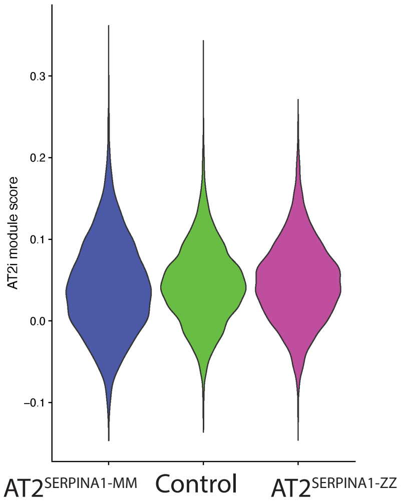

Figure S6

A) Top GO:BP terms by EnrichR of the 324 genes upregulated in iAT2 and mouse datasets.

B) Top GO:BP terms by EnrichR of the 592 genes upregulated in human lung and mouse datasets.

C) Top GO:BP terms by EnrichR of the 905 genes upregulated in human lung and iAT2 datasets.

D) Violin plot of NF- B module score across mouse genotypes.

E) Violin plot of inflammatory AT2 module score across mouse genotypes
